# Investigation on the association between serum lipid levels and periodontitis: a bidirectional Mendelian randomization analysis

**DOI:** 10.1186/s12903-023-03575-x

**Published:** 2023-11-02

**Authors:** Zhihong Chen, Jukun Song, Liszen Tang

**Affiliations:** 1 Stomatology Department, Guizhou Provinicial People’s Hospital, Guiyang, China; 2 Department of Oral and Maxillofacial Surgery, Universiti Sains Malaysia, Kota Bharu, Malaysia; 3 Department of Oral and Maxillofacial Surgery, Guizhou Medical Univerisity, Guiyang, China

**Keywords:** Periodontitis, Chronic periodontitis, Lipids, Mendelian randomization analysis

## Abstract

**Objective:**

Several research has considered the potential correlation between periodontitis and serum lipids. However, serum lipid profiles correlation with periodontitis remains largely unknown. The investigation objective was to examine periodontitis correlation with serum lipid levels using a bidirectional Mendelian randomization (MR) analysis.

**Methods:**

The study employed a bidirectional MR analysis with two samples, utilizing a freely accessible genome-wide association study (GWAS). Furthermore, the primary analysis employed the inverse variance weighted (IVW) method. To determine whether the lipid profiles were associated with periodontitis, a variety of sensitivity analyses (including MR-Egger regression, MR-PRESSO, and weighted median), as well as multivariable MR, were employed.

**Results:**

MR analysis performed by IVW did not reveal any relationship between periodontitis and low-density lipoprotein (LDL), high-density lipoprotein (HDL), triglycerides (TG), or total cholesterol (TC). It was also found that LDL, HDL, TG, and TC were not associated to periodontitis. Furthermore, the MR estimations exhibited consistency with other MR sensitivity and multivariate MR (MVMR) analyses. These results show that the correlation between serum lipid levels and periodontitis could not be established.

**Conclusion:**

The finding indicates a negligible link between periodontitis and serum lipid levels were identified, despite previous observational studies reporting a link between periodontitis and serum lipid levels.

**Supplementary Information:**

The online version contains supplementary material available at 10.1186/s12903-023-03575-x.

## Introduction

Periodontitis is a prevalent, chronic, multifactorial, infectious disease that affects the gingiva as well as the tooth’s supporting structures [1, 2]. Nearly 10% of adults and 30% of individuals above 50 have severe periodontitis [3, 4]. Periodontitis has been recognized to be linked to obesity [5, 6], as well as metabolic syndrome [7, 8]. It is well known that increased visceral fat typically leads to abnormalities in serum lipid profiles [9, 10], involving elevated levels of LDL cholesterol, HDL cholesterol, TG, and TC. Dyslipidemia is associated with various disease progress as it represents a significant cardiovascular disease (CVD) risk factor [11–14] and is involved in chronic inflammation [15, 16]. High serum lipid levels have been suggested to contribute to a pro-inflammatory condition that increases oxidative stress, resulting in hyper-reactive molecular species production imbalance with the antioxidant defense that predisposes an individual to infection [17].

Since 1999, researchers have investigated the correlation among periodontitis as well as serum lipid levels, specifically LDL, HDL, TG, and TC, considering the hypothesis that periodontitis may impact serum lipid levels [18]. Lately, various investigations have been conducted to explore the correlation between periodontitis and lipid parameters [17]. However, periodontitis impact on lipid metabolism remains debatable. Many clinical studies have reported a substantial correlation among periodontitis as well as serum lipid levels [19–21]. Conversely, several studies have documented the absence of significant associations among them [22–25]. Considering that there is still uncertainty about this association, further research on serum lipid levels and periodontitis is warranted.

The challenge of establishing causality in observational investigations may be prompted by environmental confounding or reverse causality. The utilization of genetic variants that are associated with the exposure of interest as instrumental variables to explore their effects on outcomes is an effective approach that overcomes certain constraints, commonly referred to as MR methods. At conception, genetic variants are randomly allocated, thereby reducing the impact of environmental confounding on their association with the outcome. Lately, MR methods have been utilized to explore mediated pathways [26] in which utilizing genetic variants that capture lifetime exposure assists in overcoming biases associated with measurement errors that may hinder observational studies. Therefore, the present study assessed the association between HDL, LDL, TC, and TG serum levels and periodontitis using two MR samples.

## Methods

### Ethics statement

The current investigation is a secondary analysis based on freely accessible data. All participants gave informed consent based on the original GWAS protocol, and the original GWAS authors gave ethical approval for GWAS.

### Data sources

The lipid trait data employed in this investigation were obtained from a GWAS meta-analysis carried out by the Global Lipids Genetics Consortium. The analysis examined the HDL, LDL, TG, and TC levels comprising roughly 190,000 individuals [27]. In all, 3,524, 3,060, 4,147, and 3,243 Single-nucleotide polymorphisms (SNPs) correlated with TC, HDL, LDL as well as, TG outperformed selection criterion of P-value < 5 × 10^− 8^, respectively. Details on the GWASs are presented in Table ​1.

**Table 1:** Description of lipid metabolism traits

Lipid metabolism traits	Consortium or study	Sample size	Population	Year
HDL (mg/dL)	GLGC	188,578	Trans-ethnic	2013
LDL (mg/dL)	GLGC	188,578	Trans-ethnic	2013
TC (mg/dL)	GLGC	188,578	Trans-ethnic	2013
TG (mg/dL)	GLGC	188,578	Trans-ethnic	2013

GLGC: The Global Lipids Genetics; HDL: High-density lipoprotein; LDL: Low-density lipoprotein; TC: Total cholesterol; TG: Total triglyceride

Periodontitis summarized statistics were acquired from a recent GWAS meta-analysis of gene-lifestyle interactions in Dental Endpoints (GLIDE) consortium [28] (17,353 cases; 28,210 controls). The Centers for Disease Control and Prevention and the American Academy of Periodontology evaluated similar traits through the use of probing depth measurements or self-reported data [29]. In all, 17 SNPs were identified with a threshold of *P*-value < 5 × 10^− 6^ correlated with periodontitis [30]. Moreover, present study utilized data on chronic periodontitis obtained from the FinnGen consortium (http://r4.finngen.fi/, accessed on June 1, 2022) to corroborate the findings of the associations between these exposures and serum lipid levels. The FinnGen consortium comprises 3,214 CP patients and 220,537 controls. Periodontitis was diagnosed according to code K05.3 of ICD-10 (https://risteys.finngen.fi/endpoints/K11_GINGIVITIS_PERIODONTAL#dialog-view-original-rules). The study GCST90018897, accessible at https://www.ebi.ac.uk/gwas/studies/GCST90018897 as of June 1, 2022, yielded SNPs linked to chronic periodontitis (CP) through genome-wide association. The sample consisted of 1,740 cases and 347,186 controls, and 35 SNPs with a P-value below 5 × 10^− 6^ were identified as relevant to CP and applied as instrumental variable (IV) [31] (Table 2). The criteria for periodontitis diagnosis relies on American Academy of Periodontology and European Federation of Periodontology. A patient can be identified as having periodontitis under clinical evaluation if: there is interdental Clinical Attachment Loss (CAL) present at two or more teeth that aren’t adjacent to each other, or there’s a CAL of 3 mm or more, coupled with pocket depths of 3 mm or more at two or more teeth. However, this CAL should not be attributable to other non-periodontitis-related causes which include: (a) Trauma-induced gingival recession, (b) Dental caries extending into the tooth’s cervical region, (c) CAL located on the distal side of a second molar, linked with either a malpositioned or extracted third molar, (d) An endodontic lesion that drains via the marginal periodontium, and (e) The presence of a vertical root fracture [32].

**Table 2:** Description of periodontal disease

Periodontal disease	Consortium or study	Sample size	Population	Year
Periodontal disease	GLIDE consortium	34,615	European descent	2019
Chronic periodontitis	FinnGen consortium R4 release	223,778	European	2021
Periodontal disease	GCST90018897	348,926	Trans-ethnic	2021

### Instrument selection

**Fig. 1:**
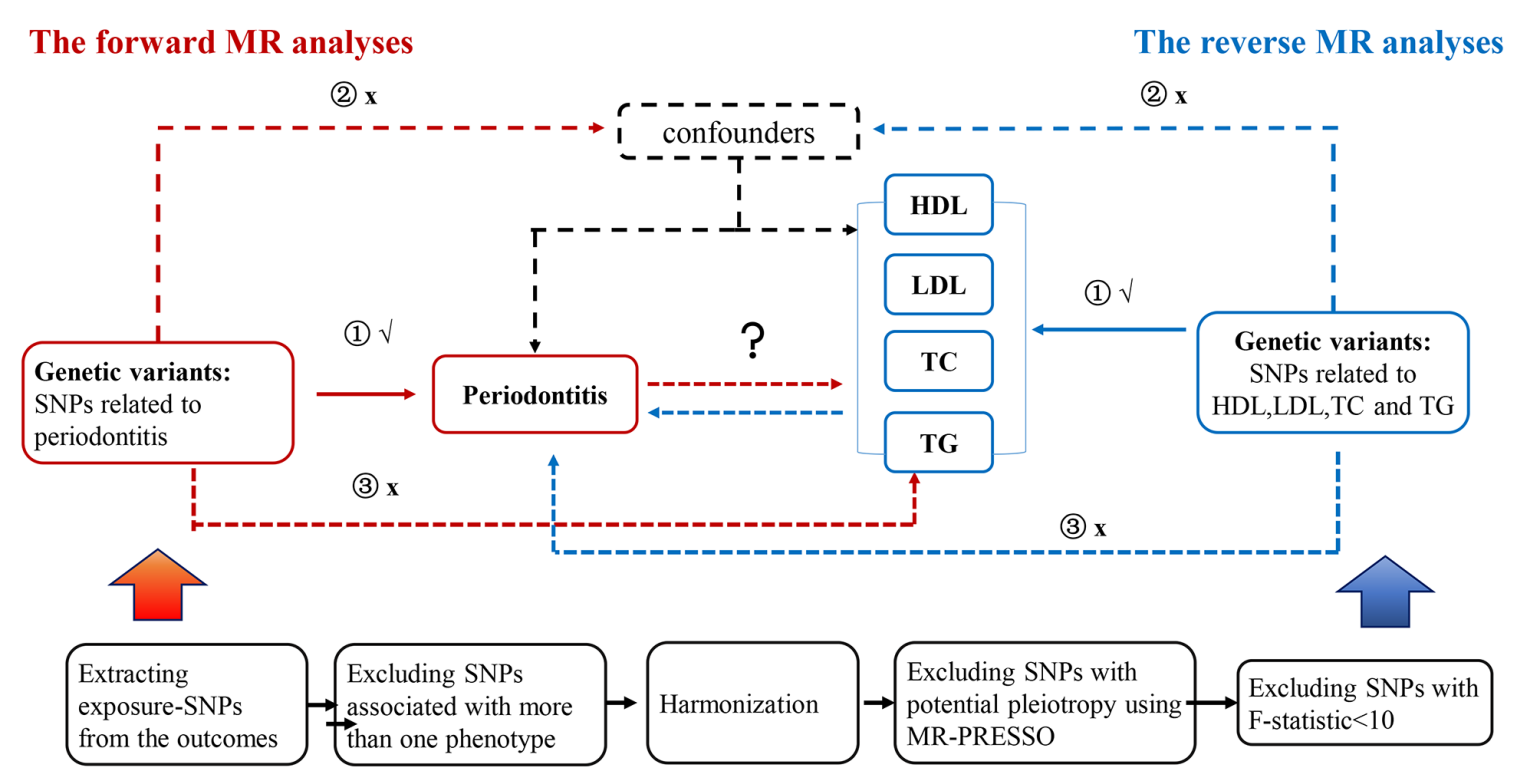
Schematic of a bidirectional two-sample MR study and three important assumptions that MR needs to satisfy

The bidirectional MR analysis was conducted using the following framework (Fig. 1). SNPs were chosen as the instruments for every exposure considered in the MR analysis based on the following criteria: (i) association hypothesis: SNPs were associated with exposure levels; (ii) independence hypothesis: There was no significant correlation observed between SNPs and the confounding factors related to both the exposure and outcome; and (iii) exclusion-limit hypothesis: SNPs affected outcomes only through exposure. A SNP was considered IV only if these three assumptions were satisfied.

### Mendelian randomization analyses

Three distinct methods of MR were employed, including the IVW, MR Egger, and weighted median, to account for the presence of variance heterogeneity and pleiotropy effects. The study employed IVW as the primary outcome, with MR-Egger along with a weighted median applied to enhance the IVW estimates due to their superior reliability, albeit less efficient, estimates across a relatively wide range of scenarios (relatively broad CI) [33, 34]. When only one SNP was available, the Wald ratio was used to estimate the causality of the outcome exposure. The MR-Egger intercept test was applied to evaluate the horizontal pleiotropy of significant SNPs. Cochran’s Q test was employed as well for heterogeneity identification. The statistical power was estimated using the online tool (https://sb452.shinyapps.io/power).

The IVs were manually searched for associations with previously reported confounders within Phenoscanner (www.phenoscanner.medschl.cam.ac.uk) to further exclude pleiotropic effects [35]. IVs related to BMI, type 2 diabetes, smoking, along with alcohol intake were closely correlated with serum lipid levels and periodontitis [36]. An additional model excluded these IVs of confounders, and MR estimates were reanalyzed to establish a more direct causal relationship. Additionally, MVMR analysis was conducted to eliminate the potential influence of correlated pleiotropy from these confounding variables. The current investigation acquired a brief overview of statistical data pertaining to type 2 diabetes (N = 69,033) from the Diabetes Genetics Replication and Meta-analysis in MVMR [37]. The Genetic Investigation of Anthropometric Traits consortium has reported a genetic association estimate for BMI, based on a sample size of 322,154 individuals [38]. Smoking statistics (N = 337,334) along with alcohol consumption statistics (N = 335,394) were compiled by Sequencing Consortium of Alcohol and Nicotine Use [39].

### Statistical analysis

All statistical analyses were conducted employing the TwoSampleMR (version 0.4.25) and MRPRESSO (version 1.0) packages available in the R (version 3.6.1) software. MR estimates were expressed as the odds ratio (OR) and its corresponding 95% confidence interval (CI), which provided an estimate of the relative risk of periodontitis caused by each standard deviation (SD) increase in lipid levels. A significant two-sided P-value was set at 0.05.

## Results

### Causal effect of lipid levels on periodontitis

**Fig. 2:**
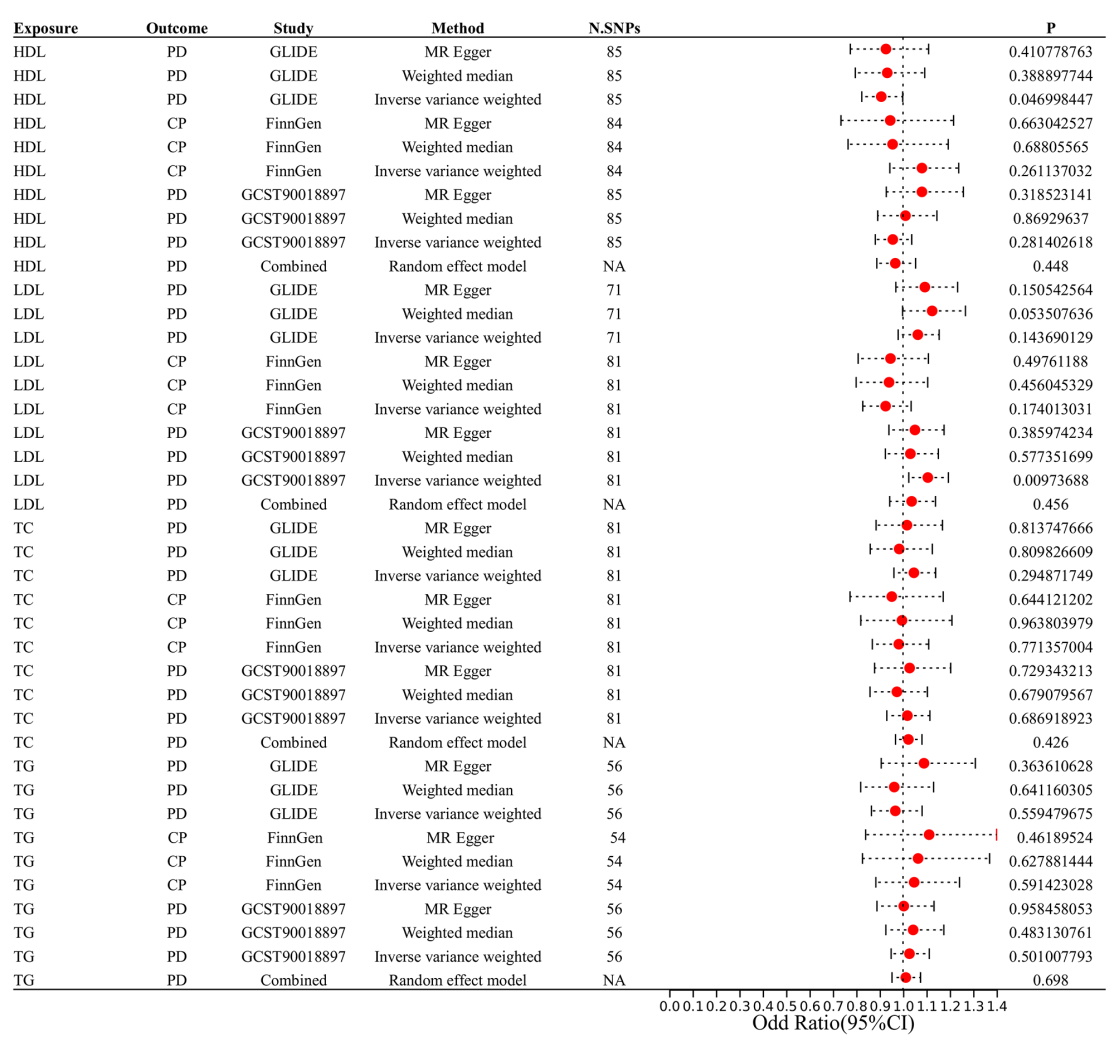
Estimated causal effect of Lipid levels on PD using different MR methods. (CI, confidence interval; OR, odds ratio; PD: periodontal disease; CP: chronic periodontitis)

The correlation between lipid profiles and periodontitis is illustrated in Table 3; Fig. 2. The IVW method demonstrated that HDL levels were associated with a 0.09% decrease in risk of periodontitis in GLIDE Consortium (HDL: N = 85 SNPs, OR: 0.91, 95% CI: 0.82–0.99, *P* = 0.04). The GLIDE Consortium study did not reveal any statistically significant relationship between periodontitis and LDL, TG, and TC lipids (LDL: N = 71 SNPs, OR: 1.06, 95% CI: 0.98–1.15, P = 0.14; TG: N = 56 SNPs, OR: 0.97, 95% CI: 0.87–1.08, P = 0.56; TC: N = 81 SNPs, OR: 1.05, 95% CI: 0.96–1.14, P = 0.29). Furthermore, the MR-Egger analyses revealed no indication of horizontal pleiotropy, as evidenced by the results presented in Table 4, where the p-value was greater than 0.05. In addition, all of the IVs exhibited F statistics that exceeded 10. The value of statistical power was between 72 and 80 (supplementary Table 1).

**Table 3:** Mendelian randomization estimates of the causal association between PD and serum lipid profile using IVW/Wald ratio method

Exposure	Outcome	OR (95% CI)	P value
HDL	PD(GLIDE)	0.91(0.82–0.99)	0.04
LDL		1.06(0.98–1.15)	0.14
TC		1.05(0.96–1.14)	0.29
TG		0.97(0.87–1.08)	0.56
HDL	CP(FinnGen)	1.08(0.94–1.24)	0.26
LDL		0.93(0.83–1.03)	0.17
TC		0.98(0.87–1.11)	0.77
TG		1.05(0.88–1.24)	0.59
HDL	PD(GCST90018897)	0.97(0.88–1.04)	0.28
LDL		1.11(1.02–1.19)	0.01
TC		1.02(0.93–1.12)	0.69
TG		1.03(0.95–1.11)	0.50
PD(GLIDE)	HDL	0.86(0.75–0.97)	0.02
	LDL	1.06(0.92–1.23)	0.40
	TC	0.99(0.87–1.14)	0.98
	TG	1.06(0.94–1.21)	0.31
CP(FinnGen)	HDL	1.03(0.95–1.12)	0.36
	LDL	0.96(0.87–1.05)	0.33
	TC	0.99(0.91–1.08)	0.92
	TG	1.02(0.95–1.11)	0.48
PD(GCST90018897)	HDL	1.02(0.97–1.08)	0.46
	LDL	1.01 (0.95–1.08)	0.74
	TC	1.03(0.96–1.09)	0.34
	TG	1.01(0.91–1.11)	0.85

**Table 4:** Sensitivity analysis of the causal association between PD and the risk of Lipid metabolism traits

Exposure	Outcome	Cochrane Q test		MR-Egger		MR-PRESSO P value
		Q value	P	Intercept	P	
HDL	PD(GLIDE)	70.33	0.85	-0.001	0.77	0.88
LDL	PD(GLIDE)	67.97	0.54	-0.002	0.53	0.62
TC	PD(GLIDE)	53.55	0.98	0.002	0.61	0.99
TG	PD(GLIDE)	50.90	0.63	-0.007	0.11	0.69
HDL	CP(FinnGen)	60.67	0.96	0.007	0.22	0.97
LDL	CP(FinnGen)	64.65	0.62	-0.002	0.69	0.67
TC	CP(FinnGen)	73.09	0.69	0.002	0.005	0.74
TG	CP(FinnGen)	56.40	0.34	-0.003	0.60	0.33
HDL	PD(GCST90018897)	87.72	0.36	-0.007	0.07	0.40
LDL	PD(GCST90018897)	75.47	0.30	0.004	0.22	0.14
TC	PD(GCST90018897)	104.47	0.06	-0.001	0.88	0.06
TG	PD(GCST90018897)	56.03	0.43	0.002	0.60	0.44
PD(GLIDE)	HDL	NA	NA	NA	NA	NA
PD(GLIDE)	LDL	NA	NA	NA	NA	NA
PD(GLIDE)	TC	NA	NA	NA	NA	NA
PD(GLIDE)	TG	NA	NA	NA	NA	NA
CP(FinnGen)	HDL	NA	NA	NA	NA	NA
CP(FinnGen)	LDL	NA	NA	NA	NA	NA
CP(FinnGen)	TC	NA	NA	NA	NA	NA
CP(FinnGen)	TG	NA	NA	NA	NA	NA
PD(GCST90018897)	HDL	0.07	0.96	-0.001	0.90	0.98
PD(GCST90018897)	LDL	0.22	0.89	-0.001	0.92	0.97
PD(GCST90018897)	TC	0.59	0.74	0.006	0.58	0.69
PD(GCST90018897)	TG	5.88	0.05	0.016	0.25	0.12

**Fig. 3:**
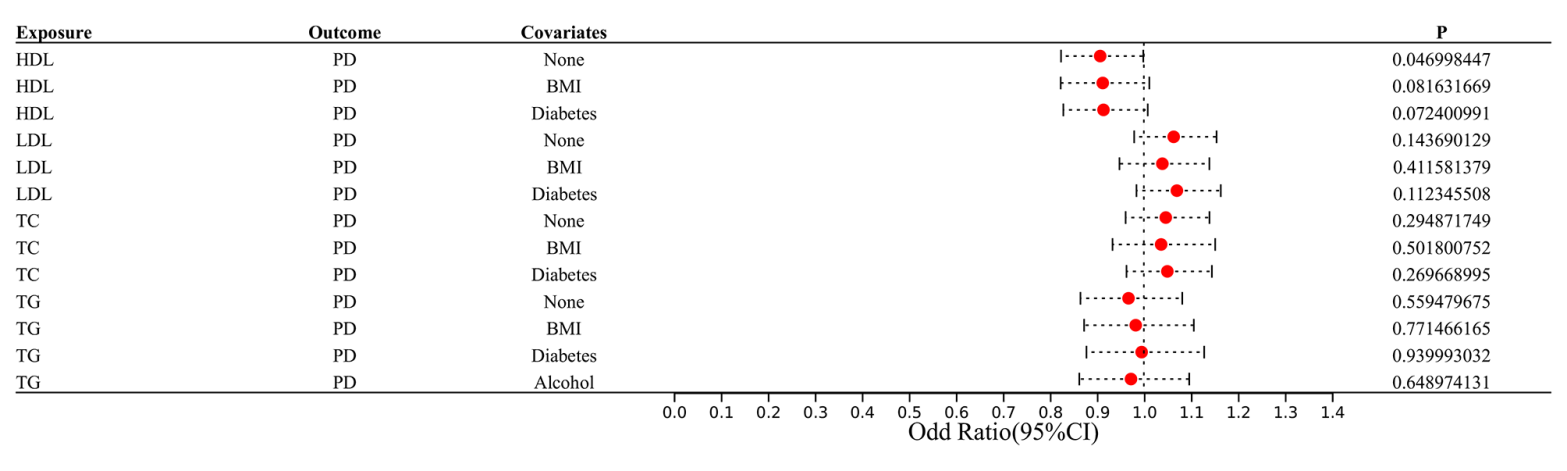
Additional Mendelian randomization analyses by removing single nucleotide polymorphisms of confounders (BMI, type 2 diabetes, smoking and alcohol intake) in the association of serum lipid levels with periodontitis/loose teeth. (CI, confidence interval; OR, odds ratio)

**Fig. 4:**
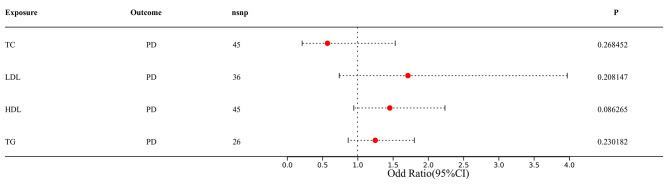
Multivariate Mendelian randomization analyses of inverse-variance weighted estimates by adjusting confounders (BMI, type 2 diabetes, smoking and alcohol intake) in the association of serum lipid levels with periodontitis in GLIDE consortium. CI, confidence interval; OR, odds ratio

The investigation mitigated the potential of correlated pleiotropy by eliminating the SNPs of the confounding variables. Subsequently, MVMR was employed to investigate the link between periodontitis and lipid levels. In Supplementary Tables 2, the features of the SNPs of confounders are provided. After the SNPs correlated with BMI, type 2 diabetes, smoking, and alcohol consumption were excluded. The additional MR analyses indicated the non-significant link between lipid levels and periodontitis persisted (Fig. 3). Additionally, MVMR estimates in the GLIDE consortium exhibited no statistically significant association between lipid levels and periodontitis after controlling for BMI, type 2 diabetes, smoking, and alcohol consumption, and the results were consistent to the univariate results (Fig. 4).

**Fig. 5:**
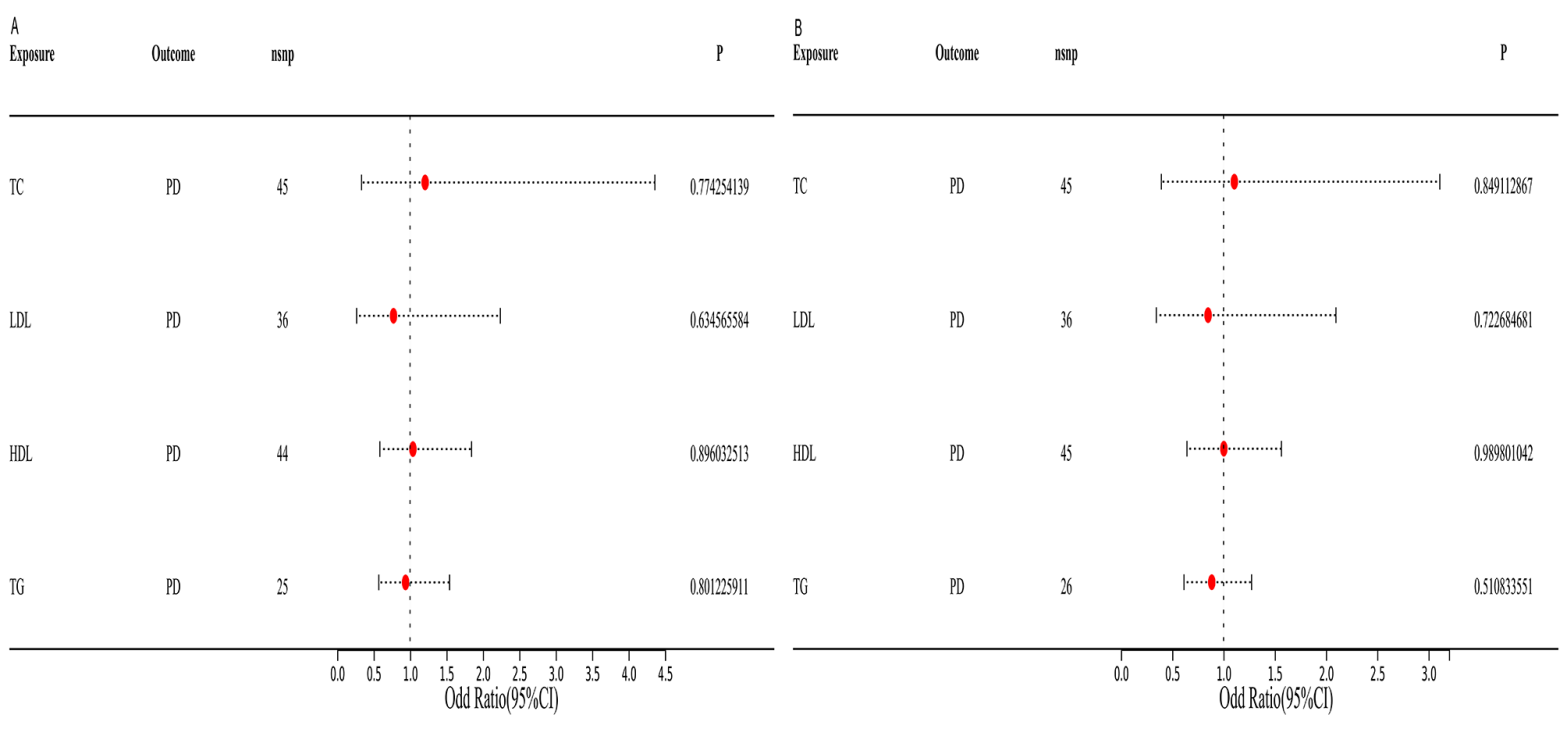
Multivariate Mendelian randomization analyses of inverse-variance weighted estimates by adjusting confounders (BMI, type 2 diabetes, smoking and alcohol intake) in the association of periodontitis with serum lipid levels (A is for Finngen consortium; B is for GCST90018897). CI, confidence interval; OR, odds ratio

In the FinnGen consortium, the relationship between lipid profiles and chronic periodontitis (CP) was further investigated, and the similar results were obtained. In the FinnGen Consortium, serum lipid levels were not related to the incidence of CP (Table 3; Fig. 2). Meanwhile, consistent findings were obtained by the GCST90018897 Consortium. In the MVMR analysis, serum lipid profiles were not associated with CP/PD in FinnGen Consortium (Fig. 5A) and GCST90018897 Consortium (Fig. 5B).

A combined analysis of GLIDE, FinnGen, and the GCST90018897 study further indicated that lipids (HDL, LDL, TG, as well as TC) and the risk of periodontitis were not found to be correlated in merged data (HDL: OR: 0.97, 95% CI: 0.89–1.05, P = 0.45; LDL: OR: 1.04, 95% CI: 0.94–1.14, P = 0.45; TC: OR: 1.02, 95% CI: 0.97–1.08, P = 0.43; TG: OR:1.01, 95% CI:0.95–1.07, P = 0.69; Fig. 2).

### Causal effect of periodontitis on lipid profiles

**Fig. 6:**
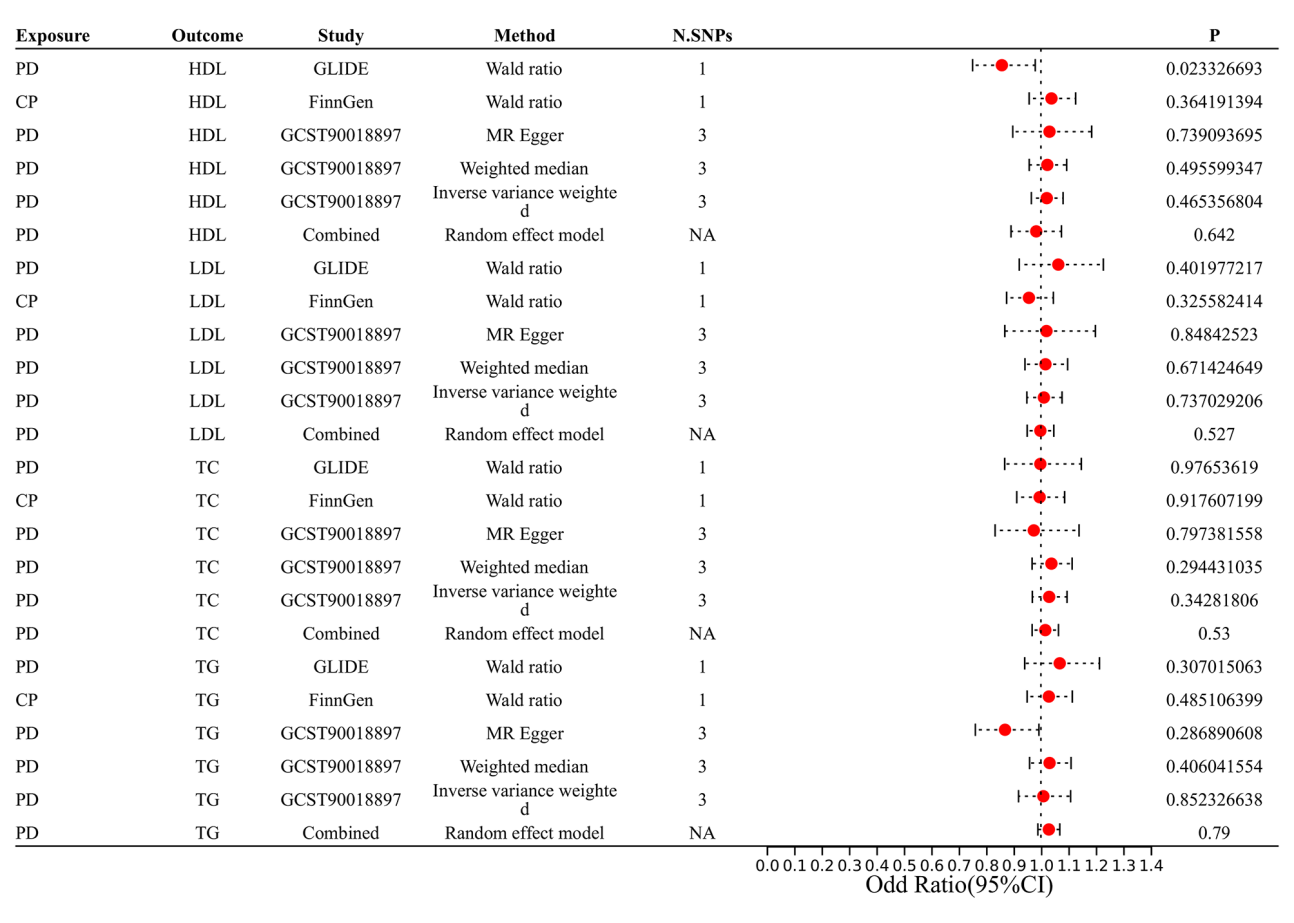
Estimated causal effect of PD on serum lipid levels using different MR methods. (CI, confidence interval; OR, odds ratio; PD: periodontal disease; CP: chronic periodontitis)

The assessment of the relationship between periodontitis and lipid profiles is illustrated in Table 3; Fig. 6. Only one SNP (rs13005050) was included in the MR analysis. Therefore, the Wald ratio was utilized to examine the correlation between periodontitis and serum lipid levels in GLIDE Consortium. Except for the HDL traits (N = 1 SNPs, OR: 0.86, 95% CI: 0.75–0.97, *P* = 0.02), the other lipid traits were not associated with periodontitis. The value of statistical power was between 70 and 86 (supplementary Table 1).

CP correlation with lipid profiles was further explored by FinnGen Consortium. To investigate the relationship between CP and serum lipid levels in the FinnGen Consortium, only one SNP (rs4880548) was included in the MR analysis. Consequently, similar results were obtained. In the meantime, GCST90018897 also produced similar findings. A combined analysis of the GLIDE, FinnGen, and GCST90018897 databases further indicated that no significant relationship existed between the periodontitis and lipids levels (HDL, LDL, TG, and TC) in pooled data (HDL: OR: 0.98, 95% CI: 0.89–1.08, P = 0.64; LDL: OR: 1.00, 95% CI: 0.95–1.05, P = 0.53; TC: OR: 1.02, 95% CI: 0.97–1.06, P = 0.53; TG: OR: 1.03, 95% CI: 0.99–1.07, P = 0.79; Fig. 6).

## Discussion

Numerous observational studies have connected dyslipidemia to periodontitis. However, the causal link remains unknown. In this investigation, two-sample MR analyses were used to comprehensively assess the causality of the association between the four lipid characteristics (HDL, LDL, TC, and TG) and the risk of developing periodontitis. The results revealed no statistically significant association between the HDL, LDL, TG, and TC levels and the periodontitis risk. Because obesity and unfavorable lipid combinations often coexist, statistically non-significant associations remained when we considered confounding factors such as BMI, diabetes, smoking, and alcohol intake. An additional model and MVMR were used to analyze, and similar results were obtained.

Whether lipid metabolism affects periodontitis risk remains controversial. A significant risk factor for developing atherosclerosis and CVD is dyslipidemia [40]. Notably, HDL cholesterol is recognized for its ability to reverse cholesterol transport, improve endothelial function, and possess antioxidant and anti-inflammatory properties [41]. A low HDL cholesterol level is an established risk factor for atherosclerosis and future CVD [42]. The extant body of literature presents divergent findings with respect to the plausible association between periodontitis and serum lipid levels. The study conducted by Korhonen and colleagues revealed a lack of statistically significant association between periodontitis and LDL cholesterol /HDL cholesterol ratio, as well as TC/HDL cholesterol ratio, within a population of Finnish individuals [43]. Saxlin et al. conducted a study on a large sample size and determined no significant correlation between serum lipid levels and periodontal infection [44]. Moreover, an earlier meta-analysis [20] has documented a significant statistical correlation between periodontitis and LDL cholesterol and TG [45]. Other studies demonstrated that lower serum HDL cholesterol levels are associated with infected gingival abscesses [46, 47]. However, because of the differences in the periodontitis assessments and the variability in the disease parameters used, substantial heterogeneity was observed in these studies. Meanwhile, periodontitis might lead to dyslipidemia. There is a possibility that these associations are caused by a combination of suboptimal health practices, including inadequate dietary patterns and insufficient oral hygiene practices [44]. The association between poor dental health habits and high serum cholesterol, TG, and HDL cholesterol supports this conclusion among the young adult Finnish population [48]. Therefore, a laboratory study is needed to investigate this mechanism in further detail.

The present study findings were consistent with those of earlier studies in which the serum lipids had no correlation with a higher probability of periodontal infection in individuals with average weight. However, in overweight individuals, there was a correlation between serum lipids and the existence of deepened periodontal pockets [44]. The deleterious effect of lipids on periodontal tissue can be ascribed to the augmented generation of pro-inflammatory cytokines and concomitant suppression of various growth factors, including platelet-derived growth factor, transforming growth factor b-1, and basic fibroblast growth factor, leading to reduced tissue regeneration [49]. It should be noted that the observed correlation between individuals who are overweight or obese and their lipid composition is likely attributed to residual confounding factors rather than indicating a real relationship among body weight and lipid composition.

Current bidirectional MR study had several advantages. First, using an MR design, the present investigation can largely simulate a randomized controlled trial within an observational context. Randomized control features are a commonly accepted methodology in studies of causality. However, they are frequently deemed costly. MR studies have the ability to effectively eliminate the confounding bias that may arise from the random assignment of SNPs during conception. In contrast to other observational research methods, the MR can evade the reverse causality effects. Second, the present study obtained the summary statistics for periodontitis to calculate the causal relationship with lipid metabolism, and the findings provide evidence for a correlation between periodontitis and lipid metabolism without establishing a causal relationship. Simultaneously, this investigation exhibited certain limitations. Firstly, the study primarily focused on individuals of European descent. The applicability of the results to other racial groups requires additional research. Secondly, while the diagnosis of periodontitis was based on the international ICD code, periodontitis was categorized into four stages according to its severity and treatment complexity. However, data on the stratification of periodontitis severity was unavailable. Thirdly, large-scale, multi-center randomized controlled studies are the gold standard for establishing causal associations. The findings of this study await validation from subsequent research. Fourthly, negative findings of lipid profiles might reflect low statistical power. The lack of statistical power might explain the inconsistent results between lipid profiles and periodontitis, which should be further examined.

In conclusion, this research results indicate that there was no statistically significant impact of periodontitis on the risk of lipid metabolism among European population.

### Electronic supplementary material

Below is the link to the electronic supplementary material.

Supplementary Material 1

Supplementary Material 2

## Data Availability

The data that support the findings of this study are available from the corresponding author upon reasonable request.
